# Carry-over body mass effect from winter to breeding in a resident seabird, the little penguin

**DOI:** 10.1098/rsos.140390

**Published:** 2015-01-21

**Authors:** Marcus Salton, Claire Saraux, Peter Dann, André Chiaradia

**Affiliations:** 1 Research Department, Phillip Island Nature Parks, PO Box 97, Cowes, Victoria 3922, Australia; 2 Institut Pluridisciplinaire Hubert Curien UMR7178 CNRS, 23 Rue Becquerel, 67087 Strasbourg, France; 3 IFREMER, UMR 212 Exploited Marine Ecosystems, Avenue Jean Monnet, BP 171, 34203 Sète Cedex, France

**Keywords:** capital-income breeding, penguin monitoring system, parental investment, timing of laying, breeding success, seabirds

## Abstract

Using body mass and breeding data of individual penguins collected continuously over 7 years (2002–2008), we examined carry-over effects of winter body mass on timing of laying and breeding success in a resident seabird, the little penguin (*Eudyptula minor*). The austral winter month of July consistently had the lowest rate of colony attendance, which confirmed our expectation that penguins work hard to find resources at this time between breeding seasons. Contrary to our expectation, body mass in winter (July) was equal or higher than in the period before (‘moult-recovery’) and after (‘pre-breeding’) in 5 of 7 years for males and in all 7 years for females. We provided evidence of a carry-over effect of body mass from winter to breeding; females and males with higher body mass in winter were more likely to breed early and males with higher body mass in winter were likely to breed successfully. Sex differences might relate to sex-specific breeding tasks, where females may use their winter reserves to invest in egg-laying, whereas males use their winter reserves to sustain the longer fasts ashore during courtship. Our findings suggest that resident seabirds like little penguins can also benefit from a carry-over effect of winter body mass on subsequent breeding.

## 2. Introduction

To determine which factors shape breeding performance of a species, studies usually focus on conditions during breeding. However, it is crucial to understand whether the parental investment into tasks associated with preparation before breeding, such as maintenance of a breeding site, acquisition of a breeding partner and allocation of food resources are linked to the offspring's chance of survival [1]. Events and processes during pre-breeding preparation stages can have an influence, i.e. have a carry-over effect, on the performance of an individual in subsequent stages of the breeding cycle [2]. In fact, carry-over effects can explain a large amount of variation in individual fitness [3].

Carry-over effects generally manifest when variations in the ability to use resources result in individuals making the transition between stages with different levels of physical condition (state), thus affecting downstream events such as reproduction or survival [3]. For instance, supplemental feeding experiments have demonstrated that increasing access to food for blue tits (*Cyanistes caeruleus*) during winter can advance laying dates and increase fledging success in the subsequent breeding season [4]. In a natural setting, proxies for resource availability, such as body condition, relate pre-migration condition of snow geese (*Chen caerulescens atlantica*) to breeding onset, with individuals of high body condition arriving at the breeding grounds earlier and laying eggs earlier in the season [5]. The advantage of carrying stored energy (e.g. in the form of fat and muscle) from wintering areas to the breeding grounds is presumably that the energy requirements for breeding may not meet the food availability on the breeding grounds [6]. Similarly, resident species can encounter important temporal variability of their prey, so that the food availability may not be sufficient during the breeding season and that stored energy could confer an important advantage during breeding. In any case, females (and males depending on the species) need an important energy budget to breed [7] which can be accumulated before breeding or acquired gradually during breeding, the capital or income models [7]. In practice, the dichotomy between income and capital breeders does not discriminate or explain fully the source of stored reserves for breeding [8–10]. Surely, there is no clear dichotomy between capital and income breeding; rather these two extreme strategies set the life-history strategy range in between which organisms have to compromise, so that most species actually exhibit a mixture of these two strategies. Conditions between breeding seasons, i.e. inter-breeding season, have the ability to shape life histories [3] as some species may have carry-over benefits of acquiring good physical condition at this time. However, the difficulty of monitoring certain species continuously throughout the inter-breeding season has limited our ability to understand the mechanisms behind carry-over effects.

Reproductive decisions begin at the inter-breeding season when individuals can decide where to breed, who to breed with and when to initiate a breeding attempt [11]. The importance of these decisions is usually species-specific, owing to differences in life-history strategies among species. Once a breeding attempt has been initiated, a parent or parents (in bi-parental species) must consider how they will balance the energy requirements of the breeding attempt (such as incubating egg(s) and feeding young) with their own energy requirements [12]. Parents who are better able to manage the trade-off between competing energy requirements are more likely to successfully raise young to independence and consequently improve their reproductive success [13]. Those that are less efficient at balancing these demands are more likely to fail reproduction [13].

In little penguins, as is the case for most birds, studies that assess mechanisms driving variation in individual fitness have mainly focused on conditions and events during the breeding season, at the most energetically demanding phase of the annual life cycle [14]. From such work, it is clear that body mass or body condition of little penguins during the breeding season is related to their timing of breeding, breeding success and is related to an individual's foraging behaviour during chick rearing [15,16]. There is limited work outside the breeding season, but in the case of little penguins, there is evidence of parents investing in future breeding during the inter-breeding season (e.g. maintaining a nest site and associating with previous and future breeding partners; [17]), which indicates potential for carry-over effects on conditions experienced at this time on subsequent breeding. Indeed, the general body condition of adult little penguins (i.e. mean adult mid-sex weight), specifically during the winter month of August (i.e. middle of the inter-breeding season), is negatively correlated with annual mean laying date in the subsequent breeding season [18]. However, until recently, establishing details about inter-individual and inter-annual elements of such carry-over effects has been limited by the ability to monitor known individuals continuously throughout their breeding cycle and across multiple years. Adult body mass does fluctuate over time [19] and has been related to changes in prey distribution and abundance [20], diet [21], energy requirements [14] and breeding chronology [15]. Further, there is evidence of individual differences in foraging ability, such that some individuals seem to be better equipped to manage periods of poor food availability than others [16]. While the mechanisms are not clear, little penguins that breed early are more likely to hatch their eggs successfully and produce heavier chicks [22]. Earlier laying is also more likely to result in relaying and, consequently, more clutches per season [17]. Although this suggests there should be selection for early breeding, the spread of laying dates can span over five months within a breeding season [17,22,23]. These variations in parent's ability to prepare for breeding support the hypothesis that there are some carry-over effects from the inter-breeding season to breeding.

Here, we used a detailed, longitudinal dataset of body mass of known individuals recorded every time the individual moved in and out of the colony over a 7 year period to further develop our understanding of the importance of winter condition on subsequent breeding. Specifically, using this dataset, we were able to separate ‘annual effects’, from within-year ‘individual effects’. We hypothesized that individuals in better condition during the inter-breeding season will be in a better position to breed earlier and perhaps attain greater reserves (or capital) in the following months leading up to breeding (i.e. individual effects). Alternatively, in years when conditions are better than average in the inter-breeding season, individuals will be more likely to breed earlier compared with other years (i.e. annual effects). The winter months of the inter-breeding season are particularly characterized by long foraging trips, far from the colony [19], low food consumption [21], high field metabolic rate [14] and low body mass [24]. This suggests penguins may be ‘working hard’ to find food resources at this particular time within the inter-breeding season. Indeed, this is likely given that prey biomass is low and prey are more sparsely distributed during winter [20]. Therefore, in this study, we focused our attention on the carry-over effects of body mass in the worse winter month (i.e. that in which they appear to be working hardest). In line with this, we expect body mass to be lower in this winter month relative to previous months, where penguins coming ashore will have recovered from moult, or after months, where penguins will be in the final stages of preparing for breeding. Finally, we tested whether body mass during winter may act as sufficient capital to buffer resource deficiencies during breeding and therefore have carry-over effects (either individual effects or annual effects) on subsequent breeding success.

## 3. Methods

### 3.1 Monitoring little penguins

The study site was located on the Summerland Peninsula on Phillip Island (38°30′S, 145°15′E), Victoria, Australia [25]. During each breeding season between 2002/2003 and 2008/2009 (2002 and 2008 for brevity), 100 artificial penguin burrows were monitored three times a week to determine individual laying dates (date first egg was laid for the season) and individual breeding success (the number of chicks fledged (0, 1 or 2) relative to the number of eggs laid (0, 1)). Birds were implanted with numbered 23 mm ISO HDX transponder (initially TIRIS, USA and later Allflex, Australia) either as chicks prior to fledging or when first encountered as an adult. Marked penguins were monitored continuously through an automated penguin monitoring system [26], which recorded presence and body mass of individual penguins every time they arrived or departed from the colony [25]. Like all penguins, little penguins have a single catastrophic moult [27] when they moult all feathers at once. Prior to moult, little penguins almost double their body mass foraging at sea before fasting for two to three weeks ashore with a sharp decrease in body mass [28]. Therefore, the attendance patterns and changes in body mass of little penguins were used to establish the start and end of moult. Adult penguins were sexed using bill depth measure [8].

At Phillip Island, the little penguin's moult typically occurs in February–March [28]. As a resident species, they continue to come ashore during austral autumn and winter months, travelling between their colonies and foraging at sea. In spring, their attendance ashore increases until laying a clutch of two eggs [25] in their second and third year after fledging [24]. Given that the moult is a predictable annual event, we defined the inter-breeding season as the period between the end of moult and subsequent laying date. As winter has the lowest productivity in temperate waters [29], we then expected the greatest disparity in foraging [21] and lowest colony attendance [17]. We identified the month of consistently lowest attendance by calculating the mean frequency of attendance per penguin (i.e. the number of individual crossings relative to the number of penguins) within each month of the year. The month of consistently lowest attendance across the 7 years (defined as ‘winter’) was subsequently a cut-off month to split the inter-breeding into three periods: moult-recovery, winter [30] and pre-breeding. The winter month was used to assess the potential for inter-breeding season carry-over effects on subsequent breeding.

### 3.2 Recording body mass

An individual's body mass, presence and identification (ID) were recorded each time little penguins arrived at the colony from January 2002 to the December 2008. The automated penguin monitoring system, designed by the Australian Antarctic Division [26], consists of a weighing platform (calculating mass to the nearest gram), a transponder reader and a computer that stores each ID and body mass and time–date. The weighing platform performs 50 weight readings per second and processes raw weight data to generate an averaged weight and its standard error at the end of each penguin's crossing. Two or more birds could cross the weighing platform at once, which can generate high or lower averages outside a little penguin weight range, followed by high standard error and therefore invalidating that given body mass measurement. We made two corrections to the body mass data to account for these errors. First, a ‘step-wise’ change in the zero weight for the weighing system was calibrated using an object of known weight. On one given day (*i*) of each month, a 1004 g object was weighed *n* times (*n* ranged 3–10) on the weighing platform and an average deviance (*x*) of the monitoring system weight from 1004 g was calculated (i.e. *x*_(*i*)_=*Σ*(1004 g—monitoring system weight_(*i*)_)/*n*_(*i*)_). All records of body mass between the day of the calibration and the previous calibration were adjusted by adding the mean deviance. Outliers were detected and excluded from the data analysis, which included records with a body mass outside the known range for little penguins at Phillip Island between 550 and 2130 g [24] and beyond the maximum change in body mass for a foraging trip between 1 and 14 days long (maximum change in weight=420 g, mean 101.1 g±8; A. Chiaradia 2003–2004, unpublished data).

We used direct body mass instead of calculating body condition. While there is variation in body size in little penguins, body mass has been shown to highly correlate with body condition (body mass adjusted for body size, *r*^2^=0.8932) producing similar patterns of body mass/condition changes [31]. Also against the use of body condition is that the addition of a linear measure of body size to calculate body condition has the potential for this index to inflate estimated variance [32]. Finally, our study had only a small sample of marked individuals with a known linear measure of body size to calculate a much smaller sample with body condition. For those reasons, we used body mass rather than body condition as a measure of an individual's physical condition between breeding seasons.

### 3.3 Data analysis

#### 3.3.1 Changes in body mass along the inter-breeding season

Three factor variables and their two- and three-way interactions were used to predict changes in body mass: inter-breeding season periods (moult-recovery, winter, pre-breeding), year (2002–2008 as a factor) and sex (male or female). Body mass was first standardized to account for the sexual body size dimorphism. Standardized body mass was calculated as an individual's body mass relative to the global mean and standard deviation of body mass for the relevant sex. Repeated measures of each individual were accounted for using linear-mixed models (LMMs), where the random effect was individual identity and the error distribution was Gaussian with an identity link function. Where there was a significant interaction, between-group effects were assessed (i.e. comparison of the inter-breeding season periods) by applying Wilcoxon single-rank tests.

#### 3.3.2 Effect of body mass on laying dates and breeding success

Both members of a pair were not always recorded by the penguin monitoring system platform in every year of the study. Rather than removing individuals from the data analysis in any given year when their partner was not recorded in that year (*n*=111 out of 519 breeding attempts), all individuals were retained and male and female effects were assessed separately. This allowed identification of sex-specific carry-over effects. This method assumes any potential carry-over effects of a parent's winter body mass on laying date and breeding success are independent of their partner's winter body mass.

Annual differences in the level of synchrony in laying date were quantified using a Levene's test, and then we test for the effect of body mass on subsequent laying date or breeding success. For that, all records of body mass within the winter month within a year were averaged (mean), so that a single but robust mean value for each individual body mass could be related to its laying date or breeding success. This prevented inflating degrees of freedom and reduced the chance of type II error if we had retained all records of body mass in the analysis. We used data in two different formats in our body mass analysis: standardized and normal (unstandardized) to address the inter-individual verses inter-annual questions. We standardized individual's body mass data against its ‘long-term’ mean (i.e. body mass over the whole study period) to look at mass change across all years, i.e. whether individual body condition and breeding success (for that individual) was related to a good or bad breeding season [33]. This addressed our inter-annual questions. The unstandardized data were used to compare inter-individual differences in winter body mass on breeding within a year as we expected a negative relationship between winter body mass of individuals and timing of laying and breeding success within each given year. Our models on carry-over effect of winter body mass on laying date used Julian laying date with a Gaussian error distribution and an identity link function. Further models investigating the carry-over effect of winter body mass on breeding success used the number of chicks fledged relative to the number of eggs laid with a binomial error distribution and a logit link function. The model addressing inter-annual differences within individuals contained standardized body mass as a fixed effect and individual identity as a random factor. The model addressing individual differences in a carry-over effect within a year contained year (as a factor), unstandardized body mass in the winter and their interaction as fixed effects, as well as individual identity as random effect. Each of these models was applied to the two response variables: laying date and breeding success (tables 1 and 3). In the second model (i.e. inter-individual differences), a significant interaction indicates different relationships between body mass and the response variable within each year. To understand the differences in the effect of body mass on laying date or breeding success within each year (i.e. individual differences within a year), a significant interaction was explored further by running separate models between body mass and the response variable for each year separately. Separate models were necessary to prevent between-year variations from confounding the effect if only a global model were used. Given that a single breeding attempt was monitored per individual within a year, these within-year models did not require a random effect for individual identity.

**Table 1.** Model selection to explain timing of laying (Julian laying date) variability in little penguins, using the factor year to prevent from inter-annual effects and highlight inter-individual effects of winter (July) body mass (BM). (The best fit model (italics) was determined according to the lowest Akaike's information criterion (AIC), supported by Akaike weights (*ω*). *k* is the number of parameters in the model. ED stands for explained deviance, i.e. the ratio of deviance explained by the model relative to the null model.)

males							
BM	year	BM : year	AICc	ΔAIC	*ω*	*k*	ED (%)
**+**	**+**		*5245.7*	*0*	*0.74*	*7*	*10*
	+		5248.6	2.91	0.17	6	10
+	+	+	5249.9	4.21	0.09	13	10
+			5801.2	555.5	0	1	0.1
			5811.2	565.5	0	0	

females							
BM	year	BM : year	AIC	ΔAIC	*ω*	*k*	ED (%)
**+**	**+**	**+**	*4198.2*	*0*	*1*	*13*	*11*
	+		4232.3	34.1	0	6	11
+	+		4233.9	35.7	0	7	10
+			4677.7	479.5	0	1	0.1
			4689.5	490.3	0	0	

All statistical analyses were computed using the R statistical framework, version 2.15.0 [34]. A maximum of likelihood-mixed model approach was used to assess the importance of the models, with model selection based on the Akaike information criterion (AIC) using both ΔAIC and Akaike weight (*ω*_*i*_) [35]. The explained deviance (ED) was estimated as the ratio of deviance explained by the model (null deviance−residual deviance) and the null model deviance and provided evidence for the amount of variability explained by the model [36]. Variables were considered significant for *p*<0.05, and means are presented with standard errors.

## 4. Results

### 4.1 Monitoring throughout the breeding cycle

Between 2002 and 2008, 173 individual penguins were recorded crossing the penguin monitoring system and were subsequently recorded breeding in the same year. Most individuals were monitored across multiple years (73%, 126 out of 173 individuals), so that a total of 519 breeding attempts were recorded. Within a year, between 59 penguins (in 2002) and 105 penguins (in 2008) were monitored (table 2). Some penguins (12 individuals; 7%) were monitored in all 7 years.

**Table 2.** Annual data from monitoring individual little penguins (IDs) crossing an automated penguin monitoring system throughout the inter-breeding season and the associated breeding chronology: length of the laying period (LLP in days), mean laying date (MLD in Julian days), and breeding success (CPP, chicks per pair). (The number of individuals that were recaptured from a previous year/s are indicated in parentheses. The number of body mass records collected by the penguin monitoring system within an inter-breeding season is divided between the moult-recovery (MR), winter (July; W) and pre-breeding (PB) periods.)

		no. body mass records					
year	no. unique IDs (recaptures)	MR	W	PB	LLP	MLD	CPP
2002	59	52	48	59	100	249	1.6
2003	67 (50)	65	64	66	45	317	1.2
2004	64 (49)	63	61	62	35	285	1.0
2005	78 (55)	69	73	75	129	284	1.2
2006	79 (59)	79	78	78	98	311	0.7
2007	72 (56)	72	70	71	108	316	1.2
2008	105 (83)	102	100	100	105	280	0.7
all years	173 (126)						

**Figure 1.**
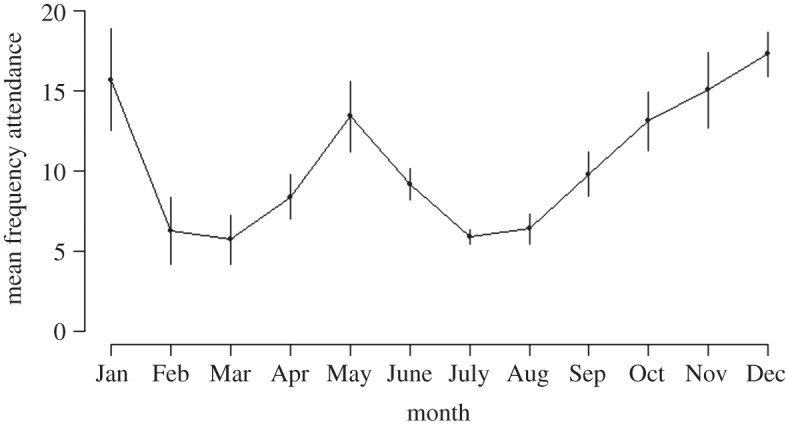
Mean frequency of attendance by individual little penguins across a penguin monitoring system at Phillip Island, Australia. Each point represents the mean (± s.e.) of the monthly frequency of attendance in each of the 7 years (2002–2008).

The number of penguins crossing the penguin monitoring system varied throughout the year. The mean frequency of attendance at the colony by an individual penguin was consistently high during the breeding months (October–January), lower during the moulting period (February and March) and consistently lowest during the winter month of July (mean 5.88 crossings±0.48; figure 1). July was therefore selected as the cut-off winter month to define the three periods of inter-breeding. Records between moult and July being ‘moult-recovery’ period and between July and laying being ‘pre-breeding’ period. Most individuals were recorded at least once within each of the three periods (79%, 411 out of 519 individuals within each year). Some individuals crossed the monitoring system up to 54 times within one of the three periods (mean 9±0.22 crossings) and up to 12 times in July (mean 2±0.09 crossings).

**Figure 2.**
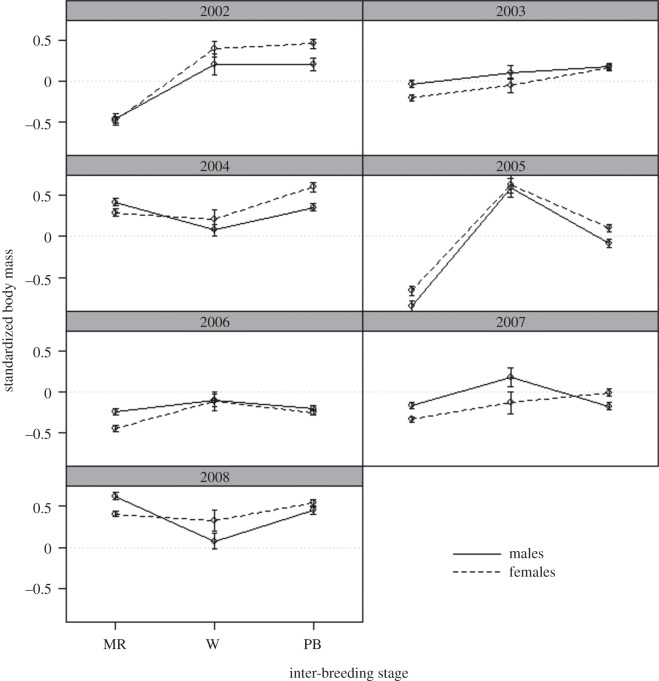
Mean standardized body mass (±s.e.) of male (solid lines) and female (dashed lines) little penguins in each inter-breeding season period (moult-recovery (MR), winter (July) (W), pre-breeding (PB)) of 7 years (2002–2008) at Phillip Island, Australia. The dotted line at zero provides a reference to the standard body mass for males and females throughout the study. Positive values indicate body mass higher than the standard and negative values are lower than standard.

The global mean body mass for all seven inter-breeding periods combined was 1119 g for females (*n* weights=7038, *N* birds=87, s.e.=2.04) and 1247 g for males (*n* weights=6614, *N* birds=86, s.e.=2.17). Throughout each inter-breeding period, body mass of males and females deviated from this global average in an inconsistent pattern among the three inter-breeding periods: moult-recovery, winter month and pre-breeding (figure 2). Within a year, the pattern of change in body mass between inter-breeding periods was also different for males and females (figure 2). Indeed, model selection identified the sex, inter-breeding period and year three-way interaction as an important variable predicting variation in body mass (log ratio=30.40, d.f.=1, *p*=0.002, Akaike weight=0.956). Males in 2005 and 2007 and females in 2005 had higher body mass in July than the moult-recovery and pre-breeding stages (Wilcoxon tests *p*<0.05; figure 2). In all years, body mass of females was higher in the pre-breeding than moult-recovery stages (Wilcoxon tests *p*<0.05). This was also the case for males in 3 years (2002, 2003, 2005), but in 2008, body mass of males was lower in the pre-breeding than moult-recovery stages and in 3 years body mass of males was not significantly different in the pre-breeding and moult-recovery stages (2004, 2006, 2007; Wilcoxon tests with *p*<0.05; figure 2).

### 4.2 Winter body mass, laying date and breeding success

There were significant variations in the synchrony of laying between years (Levene's test: *F*_6,512_=7.97, *p*<0.001). The earliest laying date recorded was 3 August (in the year 2005), and the latest was 4 January (in the year 2007). The spread of laying dates within a year was as short as 35 days in 2004 and as long as 129 days in 2005 (table 2). There were also annual differences in the mean laying dates, with mean laying date being as early as 6 September in 2002 and as late as 14 November in 2003 (table 2).

Within a year, the timing of laying was generally not related to inter-individual differences in winter body mass. The relationship between timing of laying within a year and winter body mass of the male member of the pair was consistent among years (non-significant body mass and year interaction: log ratio=7.79, d.f.=1, *p*=0.254; table 1): a linear slope between winter body mass of males and timing of laying consistently showing a non-significant difference from zero in all years (all *t*<2.00, *p*>0.05). In other words, inter-individual differences in winter body mass of males within a year did not explain differences in timing of laying within that year. The relationship between timing of laying within a year and winter body mass of the female member of the pair was consistent among all years, except 2007 (significant body mass and year interaction: log ratio=47.67, d.f.=1, *p*<0.001; table 1): a linear slope between winter body mass of females and timing of laying consistently showing a non-significant difference from zero in most years (*t*<2.00, *p*>0.05), except in 2007 where, contrary to our prediction, there was a positive effect of winter body mass of females on subsequent timing of laying (LM: slope=0.04, *t*_1,32_=2.15, *p*=0.039, *r*^2^=0.10). In other words, inter-individual differences in winter body mass of females within a year usually did not explain differences in timing of laying within that year, but in 2007, lighter females were more likely to breed earlier than heavier females.

Alternatively, when an individual's body mass is standardized against its ‘long-term’ average body mass, it is clear that annual differences in an individual's body mass explains annual differences in an individual's timing of laying. In years when males and females were heavier than their average in winter (i.e. relative to their body mass in other years), they were more likely to lay earlier in that year (LMM: males slope=−4.21, *t*_542_=−4.02, *p*<0.001, ED=0.3%, *n*=622, *N*=79, ΔAIC 14.1; females slope=−4.72, *t*_407_=−3.62, *p*<0.001, ED=0.3%, *n*=488, *N*=80, ΔAIC 11). Indeed, in the 4 years with the earlier mean laying dates (2002, 2004, 2005 and 2008), the mean standardized body mass of both males and females was above the ‘long-term’ average inter-breeding body mass for each of the sexes (figure 2).

Breeding success varied among years. Number of chicks raised per pair was highest in 2002 (1.6 chicks per pair) and lowest in 2006 (0.7 chicks per pair; table 2). The value of inter-individual differences in winter body mass of males and females to predict subsequent breeding success was inconsistent among years (male body mass and year interaction: *χ*^2^_6_ = 23.35, *p*<0.001; female body mass and year interaction: *χ*^2^_6_ = 16.59, *p*=0.011; table 3). In all years (but 2005), inter-individual differences in winter body mass of males and females did not predict subsequent breeding success within the year (females: all *z*<1.7, *p*>0.05; males: all *z*<1.8, *p*>0.05). In 2005, the variation in breeding success among individuals was attributed to their winter body mass, both in males and females (GLM: males slope=0.005, *z*=2.731, *p*=0.006, AIC=75.07, AIC null=89.315; females slope=0.006, *z*=2.209, *p*=0.027, AIC=72.46, AIC null=97.32). In 2005, the average winter body mass of males and females that successfully raised two chicks to fledging from two eggs was 1153 and 1023 g, respectively, which was greater than the winter body mass of males and females who failed to raise any chicks from two eggs (1125 and 982 g, respectively).

**Table 3.** Model selection to explain breeding success (number of chicks fledged relative to the number of eggs laid) variability in little penguins, using the factor year to prevent from inter-annual effects and highlight inter-individual effects of winter (July) body mass (BM). (The best fit model (italics) was determined according to the lowest Akaike's information criterion (AIC), supported by Akaike weights (*ω*). *k* is the number of parameters in the model. ED stands for explained deviance, i.e. the ratio of deviance explained by the model relative to the null model.)

males							
BM	year	BM : year	AIC	ΔAIC	*ω*	*k*	ED (%)
**+**	**+**	**+**	*865.5*	*0*	*0.984*	*13*	*15*
	+		874.4	8.9	0.012	6	13
+	+		876.4	10.9	0.004	7	13
+			987.1	121.6	0	1	0.3
			988.5	122.9	0	0	

females							
BM	year	BM : year	AIC	ΔAIC	*ω*	*k*	ED (%)
**+**	**+**	**+**	*662.9*	*0*	*0.80*	*13*	*22*
	+		666.8	3.89	0.12	6	20
+	+		667.5	4.59	0.08	7	20
			820.5	157.59	0	0	
+			821.2	158.32	0	1	0.2

Annual differences in breeding success were also related to annual effects on an individual's winter body mass, but for males only. In years when a male's winter body mass was heavier (relative to other years), it was more likely to raise its chicks to fledging in that year (GLMM slope=0.182, *z*=2.315, *p*=0.021, *n*=622, *N*=79, AIC=985.26, AIC null=988.44, ΔAIC 3.18). Annual differences in a female's winter body mass did not predict subsequent breeding success (GLMM: *z*=−0.865, *p*=0.387, *n*=488, *N*=80, AIC=821.75, AIC null=820.46).

## 5. Discussion

This study examined body mass variation during the inter-breeding season and the effect of winter body mass on the breeding performance of a resident seabird, the little penguin. We expected body mass to be low during winter, when colony attendance was consistently low (this study) and penguins conduct long foraging trips to the more distant foraging grounds [19]. While generally, body mass increased between moult-recovery and pre-breeding, contrary to our expectations, the winter body mass of males and females was typically equal to or higher than other inter-breeding periods. This increase in body condition in winter was probably to restore body condition lost during the previous energetically expensive breeding season and annual moult [14,37,38]. High body mass is usually associated with success during the breeding period of a bird's life cycle [9,39–43]. Here, we advance further the importance of body mass for breeding birds by providing evidence of a carry-over effect of body mass in winter with timing of breeding and reproductive success. In line with our predictions, in years where parents have higher winter body mass they were more likely to lay their eggs early, and for males (but not females), they were also more likely to breed successfully. Further, in the year of greatest asynchrony in timing of laying (2005), inter-individual differences in winter body mass of males influenced inter-individual differences in breeding success. While seabirds can meet energy requirements for reproduction by foraging concurrently during breeding (i.e. income breeders) [20,44,45], our findings support the hypothesis that they can also benefit from a carry-over effect of winter body mass on subsequent reproduction.

Winter body mass predicted laying date and breeding success and was driven by inter-annual effects on individuals, and less so on inter-individual differences within a year. In years of good resource availability, individuals in good condition are more likely to breed early. This is consistent for male and female little penguins. Male little penguins appear to be more involved in nest preparation [17], and mate guarding [25] and females are responsible for producing the clutch of eggs. It appears that winter conditions can influence the ability of males and females to achieve these roles earlier. Early laying has advantages: parents have time to make multiple attempts at breeding. Laying multiple clutches increases the potential to produce four chicks within a season [46], make a second attempt if the first clutch is not successful, or feed chicks longer in the event that conditions deteriorate for a period of time during the breeding season. This is particularly important in the marine environment of little penguins, as they rely on thermally stratified water masses to locate prey and forage more efficiently, but favourable conditions can disappear rapidly during breeding [47,48]. Similarly, inter-annual effects on the importance of winter body mass for subsequent breeding success in males, but not females, suggest resource availability during winter can vary dramatically and have significant repercussions for the entire subsequent breeding season, as opposed to just timing of laying. Alternatively, the sex difference in carry-over effect could be owing to individual variation in body size masking the signal for a carry-over effect of female body mass in winter on subsequent breeding success. But females are only slightly smaller (approx. 10%) than males [8], so variations in female body size alone could only have a small influence on sexual differences. Thus, it is more likely that the sex difference in carry-over effect of winter body mass indicates that females use reserves accumulated during winter for investment in laying [49], whereas males use their winter reserves to increase their chance of sustaining body condition throughout their longer periods ashore during courtship. Sustaining these fasts may result in sufficient energy reserves to buffer against periods of poor feeding conditions during breeding and translate into improved breeding success.

The lack of within-year differences (but 2005) might suggest either inter-individual differences in winter body mass are less important for within-year carry-over effects onto breeding or alternatively, inter-individual differences in quality may prevent a clear relationship from being apparent. Previous studies in the same location during breeding have shown that little penguins have a distinct individual quality that persists over time, regardless of sex [20]. It may be that good quality individuals are able to efficiently require resources when they are needed and therefore do not need to acquire high levels of reserves in winter in order to breed early. Or perhaps it is differences in experience, as demonstrated in breeding and foraging efficiency [22,50,51] that result in some individuals using winter reserves to improve their breeding potential, whereas others are less equipped to capitalize on this carry-over benefit. The differences only observed in 2005 illustrated that an exceptional good winter could influence subsequent breeding. Under such favourable conditions, individuals with greater winter body mass could better manage the unpredictable conditions in winter. Consequently, they would be better equipped to manage the costs of a breeding attempt and benefit from greater reproductive success. The distinction between inter-annual effects and inter-individual effects highlights the benefits of applying our analytical approach to longitudinal data on individuals.

Variation in reproduction can be explained by changes in many factors immediately prior to and during breeding, such as food availability [33] and sea surface temperature [18,23,48], pair bond duration [22], foraging performances [52], age and experience [53]. Further to that, our significant results that winter body mass explains variability in timing of breeding and reproductive success illustrate the importance of a carrying-over effect of winter body mass to the subsequent breeding. The body reserves can influence the birds' ability to sustain periods of fasting, with higher reserves allowing individuals to fast longer [54–56]. If large reserves reduce an individual's dependence on local resource availability, then even during lower resource availability these parents sustain parental duties: establish a breeding site and partner sooner, while still investing in a clutch of eggs, enabling them to breed earlier than lighter parents. If winter condition is maintained up to the egg-laying period, parents would benefit from fasting ability during incubation. The ability to fast during incubation is important in bi-parental care systems, as it can allow parents to continue incubation if the partner has an extended foraging trip [56,57]. This can improve synchronization of incubation shifts, which reduces the risk of nest failure and improves the reproductive success [25,57]. Alternatively, if lighter parents cannot sustain fasts, they may only be able to meet energetic requirements of breeding if there was high food availability close to the breeding site. One strategy to manage the risk of meeting energy demands of breeding without jeopardizing the parent's survival is to acquire and maintain good body condition while preparing for breeding, as suggested in this study.

Body mass can influence foraging behaviour of parents and subsequently limit an individual's ability to balance energy between provisioning and self-maintenance [58]. Parents with higher body mass exhibit a greater capacity to feed their chicks while maintaining their own condition, resulting in heavier chicks at fledging [16]. In little penguins, parents conduct several short trips in a row to feed the chicks often and abundantly until their body mass decreases to a point that they go out for longer trips to restore their reserves [13]. Little penguins with higher body mass may then be able to conduct a higher number of short trips than their lighter conspecifics, a behaviour increasing their reproductive success [13]. Similarly, puffins and Antarctic petrels with higher body mass feed their chicks more than those parents with lower body mass [59]. In little penguins, Zimmer *et al*. [53] found no relationship between body mass of individuals and their diving behaviour, but Saraux *et al*. [13] found body mass was an important factor influencing an individual's foraging trip duration. If body mass does influence an individual's ability to balance energy between provisioning and self-maintenance during chick provisioning, this may explain the relationship between body mass at winter and reproduction.

The link between body mass during the inter-breeding period and subsequent breeding warrants further investigation on whether there is an optimal mass to breed and whether birds skip breeding under a certain threshold as shown in blue petrels (*Halobaena caerulea*) [55]. While being heavier is generally beneficial for breeding, there are physiological and strategic adaptations to lighter body mass, including more efficient locomotion, improved foraging efficiency and reduced predation risk [60]. To determine the importance of body mass for breeding will, therefore, require knowledge of energetic costs of locomotion for a parent of varying body mass and a better understanding of strategies used by parents to accommodate changes in energy demands of the breeding attempt (e.g. higher during chick rearing). If there is an optimal body mass for breeding, then conditions influencing body mass prior to breeding appear to be important in determining timing and level of parental investment into a breeding attempt, and thus evidence of a carry-over effect between periods of the breeding cycle.

## Data accessibility

Meta data are available on National Ecological Meta Database at the Bureau of Meteorology, Australia, which can be accessed at http://www.bom.gov.au/jsp/bmrc/NEMDSearch/NemdSearch?lookup_id=81&submit=Submit.
